# Improving the Efficiency of CRISPR/Cas9-Mediated Non-Homologous End Joining Gene Knockout Using Small Molecules in Porcine Cells

**DOI:** 10.3390/biom15081132

**Published:** 2025-08-06

**Authors:** Shihao Lv, Xiaokang Xu, Sijia Yang, Mingjie Feng, Zhongyu Yuan, Xueqing Liu, Chaoqian Jiang, Jun Song, Yanshuang Mu

**Affiliations:** 1 Key Laboratory of Animal Cellular and Genetic Engineering of Heilongjiang Province, Northeast Agricultural University, Harbin 150030, China; lvshihao@neau.edu.cn (S.L.);; 2 College of Life Science, Northeast Agricultural University, Harbin 150030, China

**Keywords:** *Sus scrofa*, CRISPR/Cas9, small molecule, editing efficiency, NHEJ

## Abstract

The CRISPR/Cas9 genome editing system has emerged as an effective platform to generate loss-of-function gene edits through non-homologous end joining (NHEJ) without a repair template. To verify whether small molecules can enhance the efficiency of CRISPR/ Cas9-mediated NHEJ gene editing in porcine cells, this experiment investigated the effects of six small-molecule compounds, namely Repsox, Zidovudine, IOX1, GSK-J4, YU238259, and GW843682X, on the efficiency of CRISPR/Cas9-mediated NHEJ gene editing. The results showed the optimal concentrations of the small molecules, including Repsox, Zidovudine, IOX1, GSK-J4, YU238259, and GW843682X, for in vitro-cultured PK15 viability. Compared with the control group, the single small molecules Repsox, Zidovudine, GSK-J4, and IOX1 increased the efficiency of NHEJ-mediated gene editing 3.16-fold, 1.17-fold, 1.16-fold, and 1.120-fold, respectively, in the Cas9-sgRNA RNP delivery system. There were no benefits when using YU238259 and GW843682X compared with the control group. In the CRISPR/Cas9 plasmid delivery system, the Repsox, Zidovudine, IOX1, and GSK-J4 treatments increased the efficiency of NHEJ-mediated gene editing 1.47-fold, 1.15-fold, 1.21-fold, and 1.23-fold, respectively, compared with the control group. Repsox can also improve the efficiency of NHEJ-mediated multi-gene editing based on a CRISPR sgRNA-tRNA array. We also explored the mechanism of Repsox’s effect on the efficiency of NHEJ-mediated gene editing. The results showed that Repsox reduces the expression levels of SMAD2, SMAD3, and SMAD4 in the TGF-β pathway, indicating that Repsox can increase the efficiency of CRISPR NHEJ-mediated gene editing in porcine cells through the TGF-β pathway.

## 1. Introduction

CRISPR/Cas9 is a third-generation gene editing technology, developed after zinc finger nucleases (ZFNs) and transcription activator-like effector nucleases (TALENs), and has emerged as a leading tool in the field of genome engineering [1,2]. It has been widely applied in both basic research and therapeutic development, including gene knockout, gene knock-in, epigenetic regulation, and transcriptional control. The CRISPR/Cas9 system consists of two main components: a single-guide RNA (sgRNA) and the Cas9 nuclease. The sgRNA is engineered by fusing two naturally occurring RNAs—CRISPR RNA (crRNA) and trans-activating CRISPR RNA (tracrRNA)—to guide the Cas9 protein to a specific genomic target through base pairing [3,4,5]. Cas9 first recognizes a protospacer adjacent motif (PAM)—typically the NGG sequence—and then introduces a double-strand break (DSB) three to four base pairs upstream of the PAM site [6,7]. Following the DSB, the cellular DNA damage response is activated, initiating one of two primary repair mechanisms: non-homologous end joining (NHEJ) or homologous recombination (HR) [8]. NHEJ is a rapid but error-prone process that often results in insertions or deletions (indels), potentially disrupting the open reading frame. HR is a high-fidelity repair pathway that enables precise gene editing when a homologous DNA template is available [9,10]. Therefore, in the absence of a donor template, NHEJ-mediated repair is commonly used to generate loss-of-function mutations, making CRISPR-Cas9 a powerful tool for gene function studies and disease modeling [11,12]. In addition to gene disruption via indels, NHEJ is also exploited in targeted knock-in strategies such as obligate ligation-gated recombination (ObLiGaRe) and homology-independent targeted integration (HITI) [13,14]. In this study, we specifically focus on NHEJ-mediated gene knockout.

Pigs (*Sus scrofa*) are an important livestock species in agriculture and are used as biomedical models for human health and diseases. Genome editing enables targeted modifications at specific genomic loci to change traits in pigs, such as improving meat yields through *myostatin* (*MSTN*) gene editing and conferring resistance to porcine reproductive and respiratory syndrome virus (PRRSV) through *CD163* gene knockout [15,16]. By editing multiple genes in pigs used as human xenotransplantation donors, Massachusetts General Hospital in the United States transplanted pig kidneys modified using CRISPR/Cas9 gene editing technology with 69 gene loci into patients [17]. However, the efficiency of CRISPR/Cas9 gene knockout in pigs is still low, which limits the further application of gene-edited pigs.

Recent studies have demonstrated that small-molecule modulators can effectively enhance the efficiency of CRISPR/Cas9-mediated genome editing by targeting key components of DNA repair pathways. Stachelek et al. [18] identified a sulfonamide compound, YU238259, which specifically inhibits homologous recombination repair (HDR), thereby altering the DNA repair dynamics. Liu et al. [19] showed that the dual inhibition of HDAC1 and HDAC2 significantly enhances both non-homologous end joining (NHEJ)-mediated gene knockout and HDR-mediated gene knock-in, with HDAC inhibitors such as Entinostat and Panobinostat promoting chromatin accessibility and increasing Cas9 binding to target loci, resulting in a 1.5- to 3.4-fold improvement in editing efficiency. In a separate study, ChenYu developed a high-throughput screening platform and unexpectedly identified thymidine analogs, including zidovudine (AZT) and trifluridine, that suppress HDR and enhance NHEJ-mediated gene knockout [20]. Additionally, Mishra et al. [21] utilized a destabilized GFP-based reporter system to discover that Repsox, a TGF-β signaling inhibitor, significantly promotes NHEJ in a cell cycle-independent manner, achieving a two- to four-fold increase in editing efficiency across multiple human cell types.

To identify small molecules that enhance the CRISPR/Cas9-mediated gene knockout efficiency in porcine cells, we evaluated six candidates: Repsox, GSK-J4, IOX1, YU238259, GW843682X, and Zidovudine. This study focused on their impacts on non-homologous end joining (NHEJ) repair pathways triggered by CRISPR/Cas9. The results showed that all six compounds improved the NHEJ-mediated editing efficiency in PK15 cells, irrespective of whether Cas9 and gRNA were delivered via plasmids or alternative expression systems. Among the candidates, Repsox exhibited the most pronounced effect, increasing the editing efficiency 3.16-fold compared to the control. Moreover, Repsox enhanced the editing efficiency across multiple endogenous targets, indicating its broad potential to improve CRISPR/Cas9 outcomes in porcine cells.

## 2. Materials and Methods

### 2.1. Cell Culture

PK15 porcine kidney cells were obtained from the embryonic engineering laboratory of Northeast Agricultural University. The PK15 cells were treated at 37 °C with Dulbecco’s Modified Eagle Medium (DMEM; Gibco, #12491015, Grand Island, NY, USA) supplemented with 15% fetal bovine serum (FBS; Gibco, #A5256701, Grand Island, NY, USA) in a humidified incubator containing 5% carbon dioxide. The small molecules Zidovudine (CAS number: S2579), GW843682X (CAS number: S2880), and YU238259 (CAS number: S8379) were obtained from Selleck (Houston, TX, USA); Repsox (CAS number: HY-13012), GSK-J4 (CAS number: HY-15648B), and IOX 1 (CAS number: HY-12304) were obtained from the MedChemExpress company (Monmouth Junction, NJ, USA). These small molecules were added to the cell culture medium after the electroporation of different forms of CRISPR.

### 2.2. Design of gRNAs and Plasmid Construction

Specific guide RNAs (gRNAs) targeting GGTA1, CMAH, GHR, and B4GALNT2 were designed as follows: GGTA1-sgRNA: 5′-GCTGCTTGTCTCAACTGTAA-3′; CMAH-sgRNA: 5′-GAAGCTGCCAATCTCAAGGA-3′; GHR-sgRNA: 5′-TTCATGCCACTGGACAGATG-3′; B4GALNT2-sgRNA: 5′-AGCTCGAACACTTTCAGAGG-3′. The design of gRNAs was based on the website http://crispor.tefor.net.

To construct the CRISPR/Cas9 vector, sgRNAs and a sgRNA-tRNA array were synthesized (Genscript, Nanjing, China) and inserted into the px330 vector (Addgene, plasmid #42230) by BsmBI (New England Biolabs, NY, USA).

### 2.3. Cell Electroporation

The PK15 cells were trypsinized and neutralized by adding twice the volume of the culture medium. The number of cells was counted, and 1 × 10^6^ cells were prepared for electroporation. To prepare the ribonucleoprotein (RNP) mixture for ribonucleoprotein electrolysis, 10 µg of Cas9 protein (Genscript, #Z03470, Nanjing, China) was pre-incubated with 100 pmol of sgRNA (Genscript, #SC1838, Nanjing, China) at room temperature for 10 min. Then, Opti-MEM (Gibco, # 11058021, Grand Island, NY, USA) was used to reach the final volume of the mixture, 20 µL. For plasmid electrolysis, 5 µg of plasmid Opti-MEM was used to reach the final mixture volume, 20 µL. The cells were resuspended in the mixture, gently pipetted to maintain uniformity, and transferred to an electroporation cuvette. Electroporation was performed using a CUY21EDIT II electroporator (BEX Co., Ltd., Tokyo, Japan). For electroporation, the plasmid DNA was added to Opti-MEM to obtain a total volume of 20 μL. It was mixed with cells and set aside for use. The electroporation instrument was used to perform electroporation on the cells according to the programmed settings of 150 V, 10 ms, and 3 cycles. After electroporation, 150 μL of Opti-MEM was added to the cells and they were allowed to equilibrate at 37 °C for 5 min. Then, they were inoculated into the appropriate culture dishes and the culture was continued.

### 2.4. Cell Activity Assay

To evaluate cell viability, an Enhanced Cell Counting Kit-8 (CCK-8, #C0041) from Beyotime, Shanghai, China was used. The cells were inoculated into 96-well plates at a confluence rate of approximately 50%. After 24 h, the medium was replaced with a medium containing the test compound and incubated for another 24 h. Subsequently, 20 µL of the enhanced CCK-8 solution was added to each well, and the cells were incubated for 0.5–4 h. The absorbance was measured at 450 nm using an enzyme-linked immunosorbent assay (ELISA) reader (ThermoFisher, #1410101, Shanghai, China). The cell survival rate was calculated based on the absorbance value.

### 2.5. DNA Extraction and PCR

Genomic DNA was extracted from the cells using the Universal Genomic DNA Extraction Kit (Takara, #9765, Dalian, China), according to the manufacturer’s protocol. The target regions (200–300 bp) were amplified using high-fidelity DNA polymerase. The PCR reaction mixtures (50 µL) contained 20 ng of genomic DNA, 1× buffer, 0.5 µM of forward and reverse primers, 0.2 mM of dNTPs, and 1 U of NEBNext Q5 DNA polymerase (NEB, M0491, Beijing, China). The cycling conditions were as follows: initial denaturation at 98 °C for 5 min; 35 cycles of 95 °C for 10 s, 57 °C for 30 s, and 72 °C for 20 s. The PCR products were analyzed using 1.5% agarose gel electrophoresis and purified using a gel extraction kit (Takara, #9762, Dalian, China). The primer sequences are listed in Supplementary Table S1.

### 2.6. Deep Sequencing (Deep-Seq) and Analysis

The PCR amplicons were sent to Annoroad Gene Technology (Hangzhou, China) for deep sequencing. Libraries were constructed with unique barcoded Illumina adapters and pooled in equimolar amounts for multiplex sequencing on the Illumina MiSeq platform (Illumina, San Diego, CA, USA) using 2 × 150 bp paired-end reads. The indel frequencies were analyzed using the CRISPResso2 software (Version 2) [22].

### 2.7. Real-Time Quantitative PCR

For plasmid transfection, 5 µg of plasmid DNA was diluted in Opti-MEM to 20 µL and electroporated into 1 × 10^6^ cells, as described above. After drug treatment and culturing, the cells were harvested for RNA extraction using Trizol reagent (Takara, #9108, Dalian, China). Briefly, 1 mL of Trizol was added per 5 × 10^6^ cells, and they were homogenized and incubated for 5 min at room temperature. Then, 400 µL of chloroform (Sigma, CAS#:67-66-3, Shenyang, China) was added per 1 mL of Trizol; the samples were mixed and incubated on ice for 20 min. The samples were then centrifuged at 10,000× *g* for 15 min at 4 °C, and the aqueous phase was transferred to a new tube. RNA was precipitated by adding 500 µL of isopropanol (Sigma, CAS#:143339-58-6, Shenyang, China) per 1 mL of Trizol; samples were then incubated on ice for 10 min, centrifuged at 10,000× *g* for 10 min at 4 °C, washed with 75% ethanol (Sigma, CAS#:64-17-5, Shenyang, China), and air-dried before resuspension in RNase-free water (ThermoFisher, #10977023, Shanghai, China). cDNA was synthesized using a high-capacity reverse transcription kit (Applied Biosystems, #4368814, Shanghai, China). Quantitative PCR was performed using TB Green™ Premix Ex Taq (Takara, #CN830A, Dalian, China) on a 7500 Fast Real-Time PCR System (Applied Biosystems 7500, ThermoFisher, Shanghai, China). Relative expression was calculated using the 2^−ΔΔCT^ method with appropriate reference genes. The primer sequences are listed in Supplementary Table S2.

### 2.8. Statistical Analysis

Statistical analyses were performed using SPSS version 16.0 (SPSS Inc., Chicago, IL, USA). The data are presented as the mean ± standard error (SE) from at least three independent experiments. Statistical significance was evaluated using Student’s *t*-test, with *p* < 0.05 considered significant.

## 3. Results

### 3.1. The Effects of Different Concentrations of Small Molecules on Cell Viability

**Figure 1:**
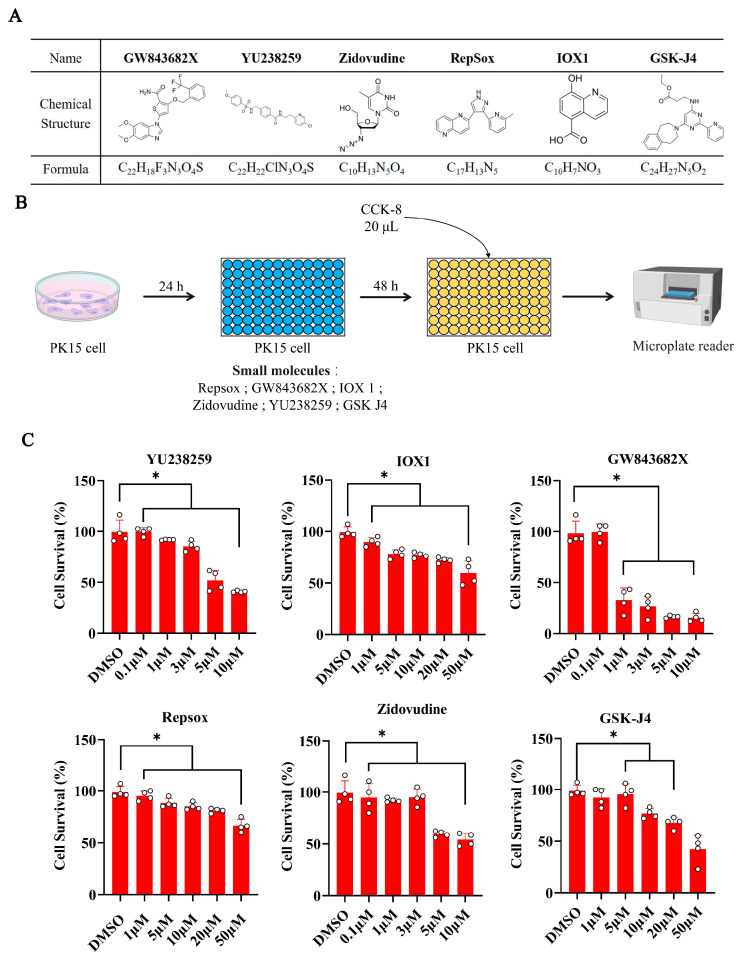
The influences of different concentrations of small molecules on the survival rates of porcine PK15 cells. (**A**) The molecular formulas and chemical structures of the six small molecules. (**B**) The experimental procedure for the cytotoxicity study of porcine PK15 cells with different concentrations of small molecules. (**C**) Cell viability assays for cell survival in PK15 cells 48 h after treatment with different concentrations of YU238259, IOX1, GW843682X, Repsox, Zidovudine, and GSK-J4. The error bars show the SEM of three replicates for PK15 cells. * *p* < 0.05.

To determine if small molecules could enhance the CRISPR/Cas9-mediated NHEJ editing efficiency in porcine renal epithelial (PK15) cells, six small molecules were selected based on the literature and the ChEMBL database [23]. The six small molecules Repsox, GSK-J4, IOX1, GW843682X, Zidovudine, and YU238259 were tested (Figure 1A). Four small molecules, including Repsox, GSK-J4, IOX1, and Zidovudine, can enhance NHEJ/alternative NHEJ. The two other small molecules, GW843682X and YU238259, can enhance the efficiency of CRISPR-mediated homologous recombination repair.

The cytotoxicity of these small molecules was evaluated by treating PK15 cells with varying concentrations for 48 h, followed by a CCK-8 assay (Figure 1B). YU238259 showed no significant cytotoxicity at 0.1–3 μM but reduced cell viability by 50% at 5 μM. IOX1 decreased viability slightly (~90%) at 1 μM. GW843682X exhibited strong cytotoxicity, reducing viability to 35% at 1 μM. Repsox showed minimal toxicity up to 20 μM but reduced viability beyond this concentration. Zidovudine had no significant effect at 1–3 μM, with the viability decreasing to 65% at 5 μM. GSK-J4 did not significantly affect viability at 1–5 μM (Figure 1C). Based on the above results and the effective doses reported in the literature, the working concentrations were determined as follows: Repsox at 10 μM, GSK-J4 at 10 μM, IOX1 at 10 μM, Zidovudine at 5 μM, YU238259 at 1 μM, and GW843682X at 0.1 μM.

### 3.2. The Effects of Different Small Molecules on the Efficiency of CRISPR RNP-Mediated NHEJ Genome Editing

**Figure 2:**
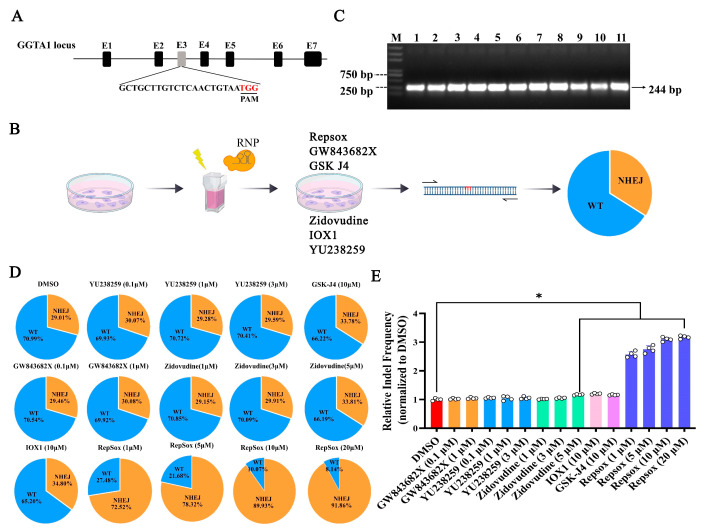
The effects of different small molecules on the efficiency of NHEJ in porcine PK15 cells. (**A**) A schematic diagram of the *GGTA1* gene structure and sgRNA target site. (**B**) A schematic of the evaluation of the genome editing efficiencies of *GGTA1* with Cas9 protein in porcine PK15 cells with small molecules. WT, wild type, unmodified; NHEJ, non-homologous end joining, modified. (**C**) PCR amplification of the *GGTA1* gene target sequence. (**D**) Genome editing efficiencies of *GGTA1* with Cas9 protein in porcine PK15 cells with different small molecules. (**E**) A histogram showing the genome editing efficiencies of *GGTA1* with Cas9 protein in porcine PK15 cells treated with different small molecules for 2 days. WT, wild type, unmodified; NHEJ, non-homologous end joining, modified. The error bars show the SEM of three replicates for PK15 cells. * *p* < 0.05.

To determine the impact of the small molecules on the efficiency of CRISPR RNP-mediated gene editing, the porcine *GGTA1* gene was targeted using an in vitro-synthesized sgRNA fragment (Figure 2A). The pre-assembled Cas9-sgRNA complex was electroporated into PK15 cells, followed by treatment with the small molecules. After 48 h, the cells were collected to assess the gene editing efficiency (Figure 2B). PCR amplification confirmed the 244 bp target sequence (Figure 2C). The sequencing analysis results showed that the percentage of indel-edited cells via NHEJ was 29.01% in the DMSO control group, 29.46–30.08% in the GW843682X (0.1, 1 μM) group, 29.59–30.07% in the YU238259 group, 29.15–33.81% in the Zidovudine (1, 3, 5 μM) group, 34.80% in the IOX1 group (10 μM), 33.78% in the GSK-J4 (10 μM) group, and 72.52–91.86% in the Repsox group. Compared to the DMSO group, the small molecules Zidovudine, GSK-J4, IOX1, and Repsox increased the NHEJ gene editing efficiency 1.17-fold, 1.16-fold, 1.20-fold, and 3.16-fold, respectively. As the small molecules GW843682X and YU238259 showed no significant improvement compared to the DMSO group, we did not conduct further experimental research on them. These results demonstrate that Zidovudine, GSK-J4, IOX1, and Repsox can all enhance NHEJ-mediated gene editing in PK15 cells, with Repsox showing the most significant effect.

### 3.3. The Effects of Different Small Molecules on the Efficiency of CRISPR/Cas9 Plasmid Vector-Mediated NHEJ Genome Editing

**Figure 3:**
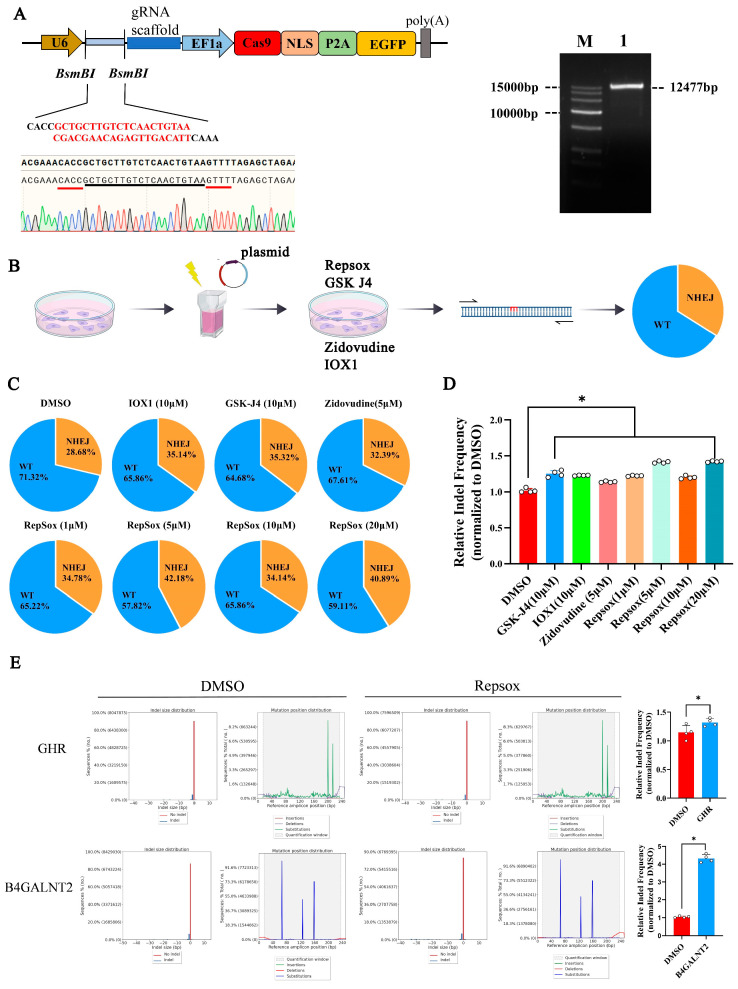
The influences of small molecules on the editing efficiency of plasmid-mediated NHEJ. (**A**) A schematic diagram of the construction of the plasmid targeting *GGTA1* and the size of the gel map of the plasmid targeting *GGTA1*. (**B**) A flowchart of the effect on genome editing efficiency of different small molecules targeting *GGTA1* in porcine PK15 cells with plasmids. (**C**) The editing efficiency for *GGTA1* in plasmid-transfected PK15 cells treated with different small molecules. (**D**) The bar chart shows the editing efficiency for the targeted gene *GGTA1* in porcine PK15 cells treated with different concentrations of small molecules in the form of plasmids for 2 days. (**E**) After treating the PK15 cells with 10 μM of Repsox, the editing efficiency for the endogenous genes *GHR* and *B4GALNT2* was assessed. WT, wild type, unmodified; NHEJ, non-homologous end joining, modified. The error bars show the SEM of three replicates for PK15 cells. * *p* < 0.05.

We constructed a CRISPR/Cas9 plasmid targeting the porcine *GGTA1* gene to investigate whether the improvement in the non-homologous end joining (NHEJ) editing efficiency using small molecules depended on the delivery mode of the CRISPR/Cas9 system (Figure 3A). The recombinant plasmids were transfected into PK15 cells via electroporation, followed by treatment with various concentrations of the small-molecule compounds: Repsox (1–20 μM), Zidovudine (5 μM), IOX1 (10 μM), and GSK-J4 (10 μM). After 48 h of culture, genomic DNA was extracted from each group, and the target site sequences were amplified for analysis (Figure 3B).

The sequencing analysis results showed that the percentage of cells that were indel-edited via NHEJ was 28.68% in the DMSO group, 32.39% in the Zidovudine group, 35.14% in the IOX1 group, 35.32% in the GSK-J4 group, and 34.14% to 42.18% in the Repsox group. Compared to the DMSO group, the small molecules Zidovudine, GSK-J4, IOX1, and Repsox increased the NHEJ gene editing efficiency 1.21-fold, 1.20-fold, and 1.19- to 1.47-fold, respectively.

To confirm the effect of Repsox on the NHEJ editing efficiency, two additional porcine genes, *B4GALNT2* and *GHR*, were assessed. The editing efficiency increased 4.2-fold for B4GALNT2 and it increased 1.23-fold for *GHR* (Figure 3E). The effect of Repsox treatment on GGTA1 editing in pig embryonic fibroblast (PEF) cells was also examined. The results indicated that Repsox treatment in PEF cells improved the GGTA1 editing efficiency (Figure S1A).

### 3.4. The Effect of Small Molecule Repsox on the Loading and Off-Target Efficiency of CRISPR/Cas9

**Figure 4:**
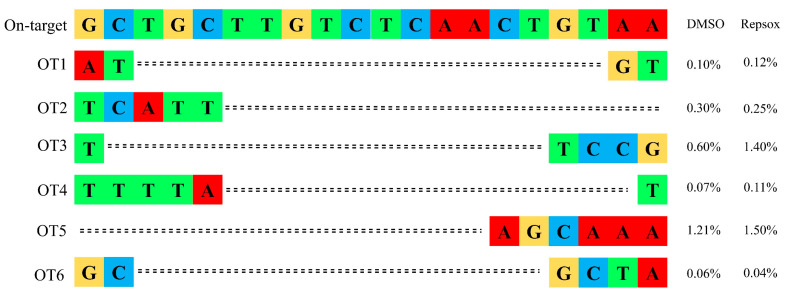
Analysis of off-target efficiency in GGTA1 using Repsox. Only the mismatched nucleotides shown in OT1–6 were compared to the target site.

Given the potential risk of off-target effects associated with CRISPR/Cas9 [23], we investigated whether the small molecule Repsox, in addition to enhancing on-target editing, affected the off-target editing efficiency. Six potential off-target sites of the *GGTA1* gene were predicted, and the editing efficiency at each off-target site was analyzed using the online tool TIDE (https://tide.nki.nl/). The results showed that Repsox treatment did not significantly alter the off-target editing efficiency compared to the control group (Figure 4).

### 3.5. The Effect of Small Molecule Repsox on the Efficiency of CRISPR/Cas9-Mediated Multi-Gene Editing

**Figure 5:**
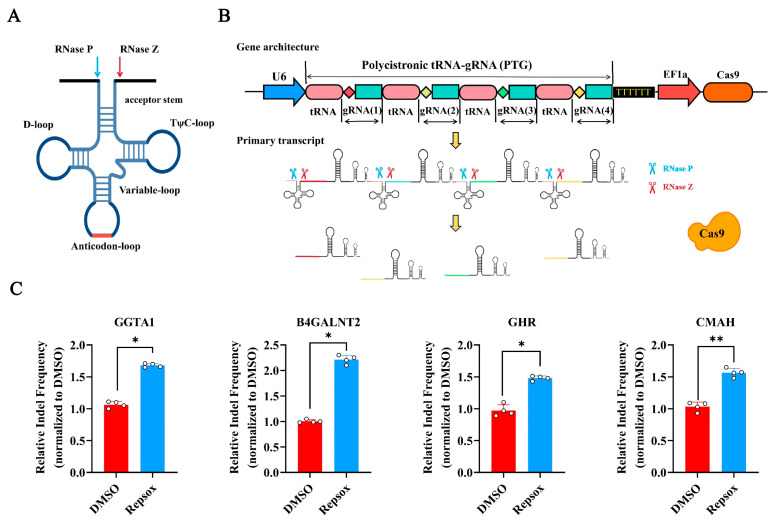
The efficiency of Repsox in the polygenic modification of porcine PK15 cells. (**A**) The secondary structure of tRNA. (**B**) A schematic diagram of CRISPR/Cas9 multi-genome editing in porcine PK15 cells using GTR-CRISPR. Primary RNA transcripts were split by porcine endogenous tRNA processing enzymes, namely endogenous RNase P and RNase Z, to generate functional guide RNAs for genome editing. (**C**) The multi-gene editing efficiency for the endogenous genes GGTA1, B4GALNT2, GHR, and CMAH after treatment with 10 μM of Repsox small molecules. * *p* < 0.05.

To explore whether Repsox could improve the CRISPR/Cas9-mediated editing efficiency across multiple genome sites simultaneously, we constructed a multi-gene-targeting CRISPR/Cas9 vector based on a tRNA-sgRNA array [24,25], targeting the four endogenous porcine genes GGTA1, B4GALNT2, GHR, and CMAH (Figure 5A,B).

PK15 cells were electroporated with the multi-gene editing vector, followed by treatment with 10 μM of Repsox for 48 h. The deep sequencing analysis results showed that, compared to the DMSO control, the Repsox treatment increased the efficiency of editing for GGTA1, B4GALNT2, GHR, and CMAH 1.70-fold, 2.30-fold, 1.50-fold, and 1.65-fold, respectively (Figure 5C). To confirm the effects of Repsox on the different cells, the multi-gene editing efficiency of Repsox in PEF cells was also studied. The results showed that Repsox could also improve the multi-gene editing efficiency in PEF cells. In PEF cells, the efficiency of editing for GGTA1, B4GALNT2, GHR, and CMAH increased by 1.87 times, 1.68 times, 1.25 times, and 2.06 times, respectively (Figure S1B). These results demonstrate that Repsox can effectively enhance the NHEJ editing efficiency of CRISPR/Cas9 at multi-genome sites simultaneously.

### 3.6. Expression Levels of Key Factors After Small-Molecule Treatment

**Figure 6:**
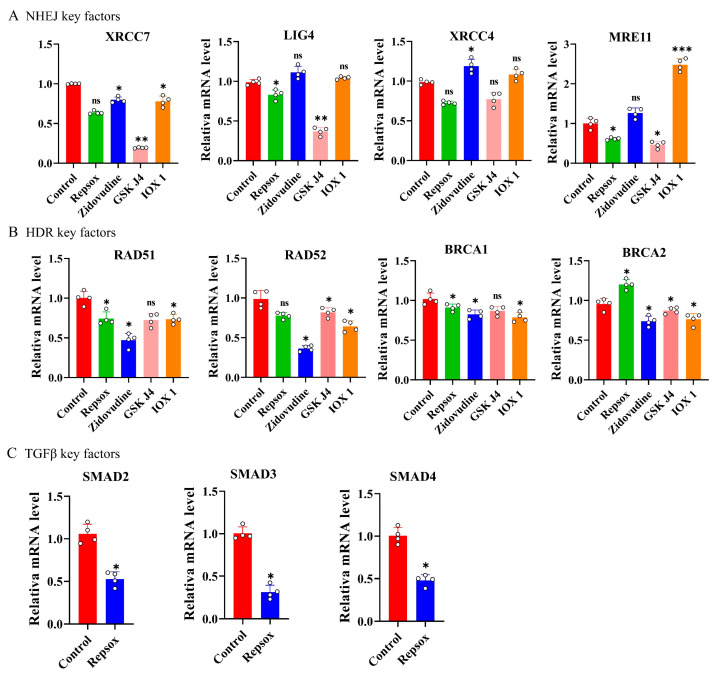
Influence of small molecules on key factors of gene expression. (**A**) Real-time fluorescence quantitative PCR result chart for NHEJ key factors. (**B**) Real-time fluorescence quantitative PCR result chart for HDR key factors. (**C**) Real-time fluorescence quantitative PCR result chart for TGF-β key factors. * *p* < 0.05.

To elucidate the molecular mechanisms by which small molecules affect gene editing, the mRNA expression levels of key factors involved in the NHEJ and HDR pathways were measured after 48 h of treatment with Repsox, IOX1, GSK-J4, and Zidovudine. The results showed that treatment with Repsox, IOX1, GSK-J4, and Zidovudine did not significantly change the mRNA levels of the core factors *XRCC7*, *LIG4*, *XRCC4*, or *MRE11* in the NHEJ pathway. Zidovudine treatment significantly upregulated only XRCC4, while downregulating *XRCC7*, *RAD51*, *RAD52*, *BRCA1*, and *BRCA2* (Figure 6A,B). This suggests that Zidovudine enhances the NHEJ-mediated gene editing efficiency, possibly by inhibiting key HDR factors [21,26].

To examine the effect of Repsox on the TGF-β signaling pathway, the mRNA expression levels of the key TGF-β signaling factors *SMAD2*, *SMAD3*, and *SMAD4* were detected. The results showed that treatment with Repsox reduced the mRNA expression levels of the key TGF-β signaling factors *SMAD2*, *SMAD3*, and *SMAD4* (Figure 6C). These findings are consistent with previous reports, indicating that Repsox enhances the CRISPR/Cas9 editing efficiency by inhibiting TGF-β signaling [17].

## 4. Discussion

Although the primary repair mechanism utilized by CRISPR/Cas9 is non-homologous end joining (NHEJ), the gene editing efficiency of CRISPR/Cas9 varies significantly depending on the cell type, with primary cells often showing low efficiencies, ranging from 2% to 25% [27]. Therefore, improving the editing efficiency remains a critical challenge. Current strategies to enhance CRISPR editing mainly focus on optimizing single-guide RNA (sgRNA) structures [28,29,30], engineering mutant Cas9 variants [31], or discovering new CRISPR/Cas systems from prokaryotes [32,33,34,35]. While these approaches are essential, small molecules offer practical advantages in terms of storage, large-scale production, and cost-effectiveness compared to protein-based methods.

Compared to previous studies targeting factors in the repair pathway or building related Cas9 fusion proteins, the advantages of NHEJ methods that use small molecules to direct DSB repair pathways lie in their ease of application in cell lines, reversibility, and rapid modes of action [36]. Therefore, the use of small-molecule compounds to enhance CRISPR/Cas9 activity seems to be a promising approach. In this study, six small molecules were tested for their ability to enhance the CRISPR/Cas9-mediated NHEJ gene editing efficiency in porcine cells. Repsox inhibits TGF-β signaling, thus inhibiting factors that are critical for the repair of DSBs, including BRCA1, ATM, and MSH2 [21]. GSK-J4 is a histone demethylase inhibitor that inhibits the JMJD3/UTX enzyme, which results in the upregulation of H3K27me3 levels [37]. IOX1 is a small-molecule drug inhibitor of HIF1α [38] that contributes to the NHEJ DNA repair pathway through the decreased expression of XRCC4. Zidovudine is a reverse transcriptase inhibitor that can reduce the homologous recombination efficiency and enhance the CRISPR-mediated genome knockout efficiency [20]. YU238259 is a novel inhibitor of the DNA polymerase θ (Polθ) that inhibits the HDR pathway but not non-homologous end joining [18]. We found that four small molecules—Repsox, GSK-J4, IOX1, and Zidovudine—significantly improved the NHEJ editing efficiency, with Repsox exhibiting the strongest enhancement. Therefore, using small molecules to improve CRISPR-mediated NHEJ gene editing remains the best choice.

Although numerous small molecules have been validated in animal models to enhance CRISPR-mediated genome editing, their clinical or routine laboratory use remains limited. The potential for adverse effects, particularly off-target mutations, immune responses, chromosomal translocations, and genetic toxicity, remains a key concern. Small-molecule modulators targeting proteins involved in the DNA damage response may improve the efficiency of CRISPR/Cas9 editing, but they can also inadvertently influence other members of the same protein family, thereby increasing the risk of unintended genomic alterations [39,40]. Moreover, the effects of small molecules on genome editing outcomes are often cell type- and cell state-dependent, likely due to variations in the activation of DNA repair signaling pathways [41]. In addition to classical non-homologous end joining (cNHEJ) and homology-directed repair (HDR), alternative pathways such as single-strand annealing (SSA), break-induced replication (BIR), and microhomology-mediated template switching also participate in double-strand break repair. These error-prone pathways tend to be more prominent in cells deficient in cNHEJ or HDR, thereby exacerbating off-target effects and reducing the HDR precision [42].

It has been reported that the inhibition of TGF-β signaling by small molecules leads to a reduction in HDR and c-NHEJ-based DSB repair but an increase in the alternative NHEJ [21,26]. In this study, we sought to investigate whether small molecules affect the expression of these genes in the NHEJ and HDR pathways, and the expression of related genes in the NHEJ and HDR signaling pathways was detected. Mechanistically, the mRNA expression of key NHEJ factors (*XRCC7*, *LIG4*, *XRCC4*, and *MRE11*) remained unchanged following treatment with these small molecules. Moreover, Repsox, GSK-J4, and IOX1 did not inhibit the mRNA levels of homologous recombination (HDR) pathway factors, whereas Zidovudine significantly suppressed critical HDR genes, which is consistent with its proposed role in favoring NHEJ over HDR. Since Repsox is an inhibitor of the TGF-β signaling pathway, we examined its effects on TGF-β-related factors (*SMAD2*, *SMAD3*, and *SMAD4*), observing significant downregulation upon treatment. Although the role of TGF-β signaling in genomic integrity is not fully understood, emerging evidence suggests that TGF-β inhibition sensitizes cells to genotoxic stress and shifts DNA double-strand break repair from canonical NHEJ and HDR towards the more error-prone alternative, the NHEJ pathway [43,44]. This shift likely underlies the enhanced gene editing efficiency observed with Repsox treatment.

We also confirmed the effects of Repsox based on different gene transfer modalities: electroporation, CRISPR-Cas9 plasmids, and Cas9-RNP. Although the efficiencies associated with each of these methods reflected the expression levels of the payload delivered and the concomitant basal editing efficiencies, the effect of Repsox remained conserved. The delivery of CRISPR/Cas9 components remains the most important factor in genome editing. A CRISPR/Cas9 multi-gene editing system based on tRNA tandem arrays was constructed to simultaneously target four genome sites. Repsox treatment increased the editing efficiency across all target genes, confirming the molecule’s utility in multiplex genome editing.

To address safety concerns, the effect of Repsox on off-target editing was assessed by amplifying and sequencing six predicted off-target sites for *GGTA1*. No significant increase in off-target editing was observed compared to the untreated controls, indicating that Repsox does not compromise Cas9 specificity. The RNP delivery mode and treatment with small molecules such as those demonstrated in this report can reduce possible off-target mutations relative to other methods, such as nucleic acid delivery by transfection, integrating viral vectors because of the short half-life of RNPs.

The advantages of using small molecules to pharmacologically guide DSB repair pathway selection towards NHEJ methods include their ease of application in cell lines, reversibility, and fast action modes. Therefore, small molecules are a promising method for the precisely control and enhancement of CRISPR/Cas9 genome editing, especially when combined with other methods. For example, CRISPR/Cas9-mediated genome editing using small molecules exhibits different enhancement or inhibition effects when delivered via the electroporation, lipid transfection, or nuclear transfection of Cas9-RNP complexes [40,45]. Meanwhile, small molecules can effectively regulate CRISPR/Cas9 activity in various cell types and other CRISPR/Cas-based systems, such as CRISPR/Cpf1, and may become a major editing tool in the future [46]. However, more work is needed to reveal the precise interaction between small molecules and Cas9 endonuclease and to expand the application of small molecules in CRISPR/Cas9 gene editing.

Despite these promising results, this study has limitations. It primarily focused on the porcine PK15 cell line, and the efficacy and safety profiles of these small molecules may vary in other cell types or species. Future studies should explore a broader range of cells to assess the generalizability. Meanwhile, we only detected the expression of related factors in NHEJ and HDR at the mRNA level. More factors should be detected at the protein level to improve our understanding of the small molecules’ mechanisms of action. Although Repsox has shown significant effects in gene editing, its mechanism has not been fully elucidated; this could be a focus of future research. Additionally, the combination of several small molecules could provide additive or synergistic effects and further improve gene editing.

## 5. Conclusions

In conclusion, Repsox significantly enhances the CRISPR/Cas9-mediated NHEJ gene editing efficiency in porcine cells, irrespective of whether Cas9 is delivered as an RNP complex or a plasmid. Repsox increased the editing efficiency for the *B4GALNT2* gene up to 4.2-fold and achieved editing efficiencies as high as 91.86% for the *GGTA1* gene. Repsox also enhances multiplex gene editing in porcine cells and improves the editing efficiency by inhibiting key components of the TGF-β signaling pathway. This study provides an efficient tool for genome editing in domesticated animals.

## Data Availability

The original contributions presented in this study are included in the article/Supplementary Materials. Further inquiries can be directed to the corresponding authors.

## Supplementary Materials

The following supporting information can be downloaded at: https://www.mdpi.com/article/10.3390/biom15081132/s1, Figure S1: NHEJ editing efficiency after treating PEF cells with Repsox; Table S1: Primers of PCR; Table S2: Primers of RT-PCR.

## References

[1] Cong L., Ran F.A., Cox D., Lin S., Barretto R., Habib N., Hsu P.D., Wu X., Jiang W., Marraffini L.A. (2013). Multiplex genome engineering using CRISPR/Cas systems. Science.

[2] Jinek M., Chylinski K., Fonfara I., Hauer M., Doudna J.A., Charpentier E. (2012). A programmable dual-RNA-guided DNA endonuclease in adaptive bacterial immunity. Science.

[3] Deltcheva E., Chylinski K., Sharma C.M., Gonzales K., Chao Y., Pirzada Z.A., Eckert M.R., Vogel J., Charpentier E. (2011). CRISPR RNA maturation by trans-encoded small RNA and host factor RNase III. Nature.

[4] Jiang F., Doudna J.A. (2017). CRISPR-Cas9 Structures and Mechanisms. Annu. Rev. Biophys..

[5] Doench J.G., Hartenian E., Graham D.B., Tothova Z., Hegde M., Smith I., Sullender M., Ebert B.L., Xavier R.J., Root D.E. (2014). Rational design of highly active sgRNAs for CRISPR-Cas9-mediated gene inactivation. Nat. Biotechnol..

[6] Iyer S., Suresh S., Guo D., Daman K., Chen J.C.J., Liu P., Zieger M., Luk K., Roscoe B.P., Mueller C. (2019). Precise therapeutic gene correction by a simple nuclease-induced double-stranded break. Nature.

[7] Zaboikin M., Zaboikina T., Freter C., Srinivasakumar N. (2017). Non-Homologous End Joining and Homology Directed DNA Repair Frequency of Double-Stranded Breaks Introduced by Genome Editing Reagents. PLoS ONE.

[8] Anzalone A.V., Koblan L.W., Liu D.R. (2020). Genome editing with CRISPR-Cas nucleases, base editors, transposases and prime editors. Nat. Biotechnol..

[9] Yeh C.D., Richardson C.D., Corn J.E. (2019). Advances in genome editing through control of DNA repair pathways. Nat. Cell Biol..

[10] Wu W.Y., Lebbink J.H.G., Kanaar R., Geijsen N., van der Oost J. (2018). Genome editing by natural and engineered CRISPR-associated nucleases. Nat. Chem. Biol..

[11] Doudna J.A., Charpentier E. (2014). Genome editing. The new frontier of genome engineering with CRISPR-Cas9. Science.

[12] Doudna J.A. (2020). The promise and challenge of therapeutic genome editing. Nature.

[13] Maresca M., Lin V.G., Guo N., Yang Y. (2013). Obligate ligation-gated recombination (ObLiGaRe): Custom-designed nuclease-mediated targeted integration through nonhomologous end joining. Genome Res..

[14] Suzuki K., Tsunekawa Y., Hernandez-Benitez R., Wu J., Zhu J., Kim E.J., Hatanaka F., Yamamoto M., Araoka T., Li Z. (2016). In Vivo genome editing via CRISPR/Cas9 mediated homology-independent targeted integration. Nature.

[15] Song R., Wang Y., Zheng Q., Yao J., Cao C., Wang Y., Zhao J. (2022). One-step base editing in multiple genes by direct embryo injection for pig trait improvement. Sci. China Life Sci..

[16] Xu K., Zhou Y., Mu Y., Liu Z., Hou S., Xiong Y., Fang L., Ge C., Wei Y., Zhang X. (2020). CD163 and pAPN double-knockout pigs are resistant to PRRSV and TGEV and exhibit decreased susceptibility to PDCoV while maintaining normal production performance. Elife.

[17] Anand R.P., Layer J.V., Heja D., Hirose T., Lassiter G., Firl D.J., Paragas V.B., Akkad A., Chhangawala S., Colvin R.B. (2023). Design and testing of a humanized porcine donor for xenotransplantation. Nature.

[18] Stachelek G.C., Peterson-Roth E., Liu Y., Fernandez R.J., Pike L.R., Qian J.M., Abriola L., Hoyer D., Hungerford W., Merkel J. (2015). YU238259 Is a Novel Inhibitor of Homology-Dependent DNA Repair That Exhibits Synthetic Lethality and Radiosensitization in Repair-Deficient Tumors. Mol. Cancer Res..

[19] Liu B., Chen S., Rose A., Chen D., Cao F., Zwinderman M., Kiemel D., Aissi M., Dekker F.J., Haisma H.J. (2020). Inhibition of histone deacetylase 1 (HDAC1) and HDAC2 enhances CRISPR/Cas9 genome editing. Nucleic Acids Res..

[20] Yu C., Liu Y., Ma T., Liu K., Xu S., Zhang Y., Liu H., La Russa M., Xie M., Ding S. (2015). Small molecules enhance CRISPR genome editing in pluripotent stem cells. Cell Stem Cell.

[21] Mishra T., Bhardwaj V., Ahuja N., Gadgil P., Ramdas P., Shukla S., Chande A. (2022). Improved loss-of-function CRISPR-Cas9 genome editing in human cells concomitant with inhibition of TGF-beta signaling. Mol. Ther.-Nucleic Acids.

[22] Clement K., Rees H., Canver M.C., Gehrke J.M., Farouni R., Hsu J.Y., Cole M.A., Liu D.R., Joung J.K., Bauer D.E. (2019). CRISPResso2 provides accurate and rapid genome editing sequence analysis. Nat. Biotechnol..

[23] Bento A.P., Gaulton A., Hersey A., Bellis L.J., Chambers J., Davies M., Kruger F.A., Light Y., Mak L., McGlinchey S. (2014). The ChEMBL bioactivity database: An update. Nucleic Acids Res..

[24] Dong F., Xie K., Chen Y., Yang Y., Mao Y. (2017). Polycistronic tRNA and CRISPR guide-RNA enables highly efficient multiplexed genome engineering in human cells. Biochem. Biophys. Res. Commun..

[25] Xie K., Yang Y. (2019). A Multiplexed CRISPR/Cas9 Editing System Based on the Endogenous tRNA Processing. Methods Mol. Biol..

[26] Li G., Zhang X., Zhong C., Mo J., Quan R., Yang J., Liu D., Li Z., Yang H., Wu Z. (2017). Small molecules enhance CRISPR/Cas9-mediated homology-directed genome editing in primary cells. Sci. Rep..

[27] Mali P., Yang L., Esvelt K.M., Aach J., Guell M., DiCarlo J.E., Norville J.E., Church G.M. (2013). RNA-guided human genome engineering via Cas9. Science.

[28] Ran F.A., Hsu P.D., Lin C.Y., Gootenberg J.S., Konermann S., Trevino A.E., Scott D.A., Inoue A., Matoba S., Zhang Y. (2013). Double nicking by RNA-guided CRISPR Cas9 for enhanced genome editing specificity. Cell.

[29] Ryan D.E., Taussig D., Steinfeld I., Phadnis S.M., Lunstad B.D., Singh M., Vuong X., Okochi K.D., McCaffrey R., Olesiak M. (2018). Improving CRISPR-Cas specificity with chemical modifications in single-guide RNAs. Nucleic Acids Res..

[30] Fu Y., Sander J.D., Reyon D., Cascio V.M., Joung J.K. (2014). Improving CRISPR-Cas nuclease specificity using truncated guide RNAs. Nat. Biotechnol..

[31] Perez-Pinera P., Kocak D.D., Vockley C.M., Adler A.F., Kabadi A.M., Polstein L.R., Thakore P.I., Glass K.A., Ousterout D.G., Leong K.W. (2013). RNA-guided gene activation by CRISPR-Cas9-based transcription factors. Nat. Methods.

[32] Abudayyeh O.O., Gootenberg J.S., Essletzbichler P., Han S., Joung J., Belanto J.J., Verdine V., Cox D.B.T., Kellner M.J., Regev A. (2017). RNA targeting with CRISPR-Cas13. Nature.

[33] Abudayyeh O.O., Gootenberg J.S., Konermann S., Joung J., Slaymaker I.M., Cox D.B., Shmakov S., Makarova K.S., Semenova E., Minakhin L. (2016). C2c2 is a single-component programmable RNA-guided RNA-targeting CRISPR effector. Science.

[34] Amrani N., Gao X.D., Liu P., Edraki A., Mir A., Ibraheim R., Gupta A., Sasaki K.E., Wu T., Donohoue P.D. (2018). NmeCas9 is an intrinsically high-fidelity genome-editing platform. Genome Biol..

[35] Edraki A., Mir A., Ibraheim R., Gainetdinov I., Yoon Y., Song C.Q., Cao Y., Gallant J., Xue W., Rivera-Perez J.A. (2019). A Compact, High-Accuracy Cas9 with a Dinucleotide PAM for In Vivo Genome Editing. Mol. Cell..

[36] Yang H., Ren S., Yu S., Pan H., Li T., Ge S., Zhang J., Xia N. (2020). Methods Favoring Homology-Directed Repair Choice in Response to CRISPR/Cas9 Induced-Double Strand Breaks. Int. J. Mol. Sci..

[37] Kruidenier L., Chung C.W., Cheng Z., Liddle J., Che K., Joberty G., Bantscheff M., Bountra C., Bridges A., Diallo H. (2012). A selective jumonji H3K27 demethylase inhibitor modulates the proinflammatory macrophage response. Nature.

[38] Schiller R., Scozzafava G., Tumber A., Wickens J.R., Bush J.T., Rai G., Lejeune C., Choi H., Yeh T.L., Chan M.C. (2014). A cell-permeable ester derivative of the JmjC histone demethylase inhibitor IOX1. ChemMedChem.

[39] Di Stazio M., Foschi N., Athanasakis E., Gasparini P., d’Adamo A.P. (2021). Systematic analysis of factors that improve homologous direct repair (HDR) efficiency in CRISPR/Cas9 technique. PLoS ONE.

[40] Kagita A., Lung M.S.Y., Xu H., Kita Y., Sasakawa N., Iguchi T., Ono M., Wang X.H., Gee P., Hotta A. (2021). Efficient ssODN-Mediated Targeting by Avoiding Cellular Inhibitory RNAs through Precomplexed CRISPR-Cas9/sgRNA Ribonucleoprotein. Stem Cell Rep..

[41] Hirakawa M.P., Krishnakumar R., Timlin J.A., Carney J.P., Butler K.S. (2020). Gene editing and CRISPR in the clinic: Current and future perspectives. Biosci. Rep..

[42] Hu Q., Lu H., Wang H., Li S., Truong L., Li J., Liu S., Xiang R., Wu X. (2019). Break-induced replication plays a prominent role in long-range repeat-mediated deletion. EMBO J..

[43] Le B.V., Podszywalow-Bartnicka P., Maifrede S., Sullivan-Reed K., Nieborowska-Skorska M., Golovine K., Yao J.C., Nejati R., Cai K.Q., Caruso L.B. (2020). TGFbetaR-SMAD3 Signaling Induces Resistance to PARP Inhibitors in the Bone Marrow Microenvironment. Cell Rep..

[44] Liu S., Khan A.R., Yang X., Dong B., Ji J., Zhai G. (2021). The reversal of chemotherapy-induced multidrug resistance by nanomedicine for cancer therapy. J. Control. Release.

[45] Okamoto S., Amaishi Y., Maki I., Enoki T., Mineno J. (2019). Highly efficient genome editing for single-base substitutions using optimized ssODNs with Cas9-RNPs. Sci. Rep..

[46] Ma X., Chen X., Jin Y., Ge W., Wang W., Kong L., Ji J., Guo X., Huang J., Feng X.H. (2018). Small molecules promote CRISPR-Cpf1-mediated genome editing in human pluripotent stem cells. Nat. Commun..

