# Supplementary material for: Improving the Efficiency of CRISPR/Cas9-Mediated Non-Homologous End Joining Gene Knockout Using Small Molecules in Porcine Cells
Source: Biomolecules. 2025 Aug 6;15(8):1132. doi: 10.3390/biom15081132 (PMC12384319; doi:10.3390/biom15081132)
Supplement: Supplementary file 1 [file biomolecules-15-01132-s001.zip › biomolecules-3742849-supplementary.pdf]

Supplementary Materials

A

PEF cells :

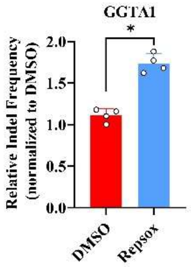

B

PEF cells :

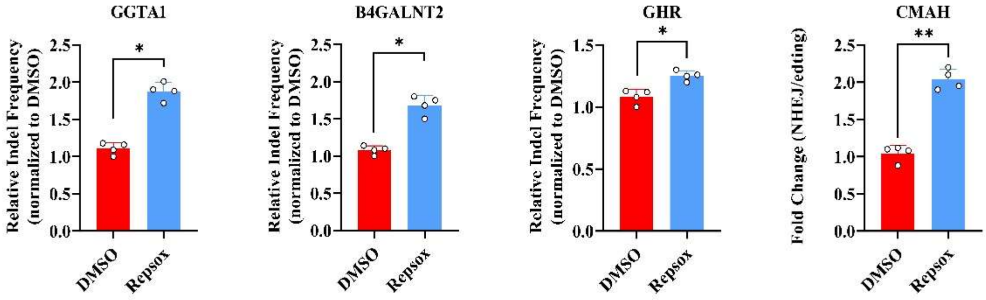

Figure S1. NHEJ editing efficiency after treating PEF cells with Repsox. (**A**) The editing efficiency of single-gene GGTA1 after treating PEF cells with 10 μM Repsox. (**B**) The multi-gene editing efficiency of endogenous multiple genes GGTA1, B4GALNT2, GHR and CMAH in PEF cells treated with 10μM Repsox. * $p < 0.05$; ** $p < 0.01$.

Table S1. Primers of PCR.

Primer ID	Sequence (5'–>3')
ggta1-244F	CCATATTCCACTCTGGGTGTATTT
ggta1-244R	GTGAATCCTACTTTTAATGCAAGC
CHAH-326F	GCTCAGGGATTCTCTCTTAA
CHAH-326R	CTTGGTGCTTGCACATG
GHR-249F	CCTCCTGATCTCATGCCTTG
GHR-249R	TGAGTCATATGCAATAGTAG
B4GALNT2- F	CCGGACCGAGCTTGCGGCTCC
B4GALNT2-R	AAGAGGGCATTCCCTCCCC

Table S2. Primers of RT-PCR.

q-SMAD2-F	TTGAGCCACAGAGTAATT
q-SMAD2-R	AAGAGTAGTAGGCGATAGTT
q-SMAD3-F	AACATTCCTGTGGCTTCC
q-SMAD3-R	CATTCTGCTGCTGTCCTG
q-SMAD4-F	CTGACCTTCACAGCCACCA
q-SMAD4-R	ACGCAGACCTCGTCCTTC
q- LIG4-F	GCCGCTATCGCAGACATTG
q- LIG4-R	GCCATCATCTCACCATCAAGG
q-XRCC4-F	AGCATTGGTGTCAGGAGCAG
q-XRCC4-R	GGTGTCCAGGCAGTAACAAATAAG
q-XRCC7-F	GCAGAATTATTCCAGCATTGATG
q-XRCC7-R	TGGGACGATAAATTACCTTGTTTG
q-MRE11-F	ATGACTTCCTTGACCTTGTTATCTG
q-MRE11-R	ATGTTTCTTTACCGCTTCTCCTG

q-BRCA1-F	ACGCCACTCTCAACTTCTG
q-BRCA1-R	CAAGCCTGATGCCACAATAG
q-BRCA2-F	GAGGAGGAGGAGGAGGATG
q-BRCA2-R	ATCTGTTAGTTCTGCTGTGTTC
q-RAD51-F	CGTTCAACACAGACCACCAG
q-RAD51-R	GCAAGTCGCAGAAGCATCC
q-RAD52-F	CTACTGGTGGCAACTCTGTATTATG
q-RAD52-R	ACCCTGTGACCCTCAATGTAAC
